# Supplementary material for: Dosage compensation of Z sex chromosome genes in avian fibroblast cells
Source: Genome Biol. 2023 Sep 20;24:213. doi: 10.1186/s13059-023-03055-z (PMC10510239; doi:10.1186/s13059-023-03055-z)

Additional file 1

Supplementary Figure Legends

Fig. S1 Experimental design and the data analysis pipeline. **A:** Embryonic fibroblast cell morphology from three representative bird species. **B:** CAGE (cap analysis of gene expression) library preparation and processing steps. In total 40 samples from seven bird species with male and female samples in six replicates (three embryos x two culture conditions), except for the finch (4 replicates with three female samples and one male sample) were sequenced on Illumina Hiseq2500 with an average depth of 20M reads. After quality assessment reads were trimmed by quality scores, against adapters and rRNA sequences. Trimmed CAGE reads were aligned to reference genome assemblies (**Table S2**) and processed with PromoterPipeline (https://github.com/Population-Transcriptomics/C1-CAGE-preview) scripts to obtain CAGE peaks with expression values. CAGE peaks were associated to nearby genes and classified into "promoters" or "not-promoters" (M&M).

Fig. S2 Correlation between CAGE replicates. Pearson correlation for CAGE samples and replicates calculated on log10(TPM) values for all CAGE peaks.

Fig. S3 Correlation between CAGE-seq and RNA-seq methods in birds. Scatter plots show log2(cumulative TPM) values for CAGE and log2(FPKM) for RNAseq with expression threshold for log2values > 0.1. Two-sided Pearson's test results are shown on top of the scatterplots for each species.

Fig. S4 Peak distribution and model efficiency estimation. A: CAGE peak distribution with regard to gene models. For turkey, there are two assemblies used: melGAl5 and MGAL_WU_HG_1.0 (labeled as "turkey"). For emu: droNov1 and ZJU1.0 (labeled as "emu"). Notably, significant number of peaks located in distal intergenic regions in both turkey assemblies, in peacock and ZJU1.0 emu assemblies. This is probably associated with gene model quality. We applied a sequence structure-based classification for CAGE peaks (TSSClassifier, M&M) using sequences of CAGE peaks located in known promoter regions as training set for the model. **B:** ROC analysis for the model efficiency estimation. This approach allowed us to rescue up to 17.8% CAGE peaks (**Table S2**). CAGE peaks localized in known promoter regions (3 kb from gene model start site) and remaining peaks classified as promoters were used in the rest steps of the analysis. **C:** CAGE peaks and promoters validation by RNAseq with 1FPKM threshold for positive genes.

Fig. S5 Sample MDS plots and hierarchical clustering. A: MDS plots for bird fibroblast cells using TPM (tags per million) counts of top 500 highly expressed genes. **B:** Expression profiles of CAGE peaks located on sex chromosomes. Hierarchical clustering of samples results in clear separation of male and female fibroblast cells. There is an obvious lack of chrW gene expression in male samples. In quail and turkey, chrW is not available, but the pattern was observed for chrZ genes. In peacock, expression of genes located on scaffolds similar to chicken sex chromosomes was enough for sample clustering into males or females (scaffold227_len202630_cov0 – chrZ, scaffold1585_len299260_cov0 – chrW defined by best blastn match).

Fig. S6 Expression profiles for gametologue genes defined by blast between chrW and chrZ genes. A: Chicken (GRCg6a) - 26 out of 30 gametologues are differentially expressed between male and female fibroblast cells (*ZFR*, *ZSWIM6*, *SUB1* and *MIER3* are not differentially expressed), **B:** Emu (GCA_016128335.1 ZJU1.0) - among 30 gametologues, there are three non-differentially expressed genes

g14360.t1 (*ANXA1*), g12622.t1 (*PSAT1*) and g12624.t1 (*TLE4Z1*). **C:** Duck (GCF_015476345.1 ZJU1.0) - there are 17 non-differentially expressed gametologue genes out of 50 determined by blast: *MAP1B, CHD1, RICTOR, ZFR, RASA1, PIAS2, UBAP1, SMAD2, KIF2A, SMAD7, LOC101794123, DNAJB5, GPBP1, GOLPH3, FAM219A, RPL37* and *IER3IP1*. **D:** Zebra finch (bTaeGut2.pat.W.v2) - out of 37 gametologues detected by blast there are 19 non-differentially expressed genes: *MAP1B, CHD1, ZFR, RASA1, TNPO1, KIF2A, SREK1, SNX18, ARRDC3, ZNF131, FCHO2, LOC100229438, GOLPH3, CDC37L1, SPIN1, UBE2R2, ZFAND5, SUB1* and *CZH18orf32*.

Fig. S7 Genomic views. A: Emu CAGE expression in fibroblast cells on chrZ sex determining region (SDR) and pseudoautomosal regions (PAR); **B:** CAGE expression in fibroblast cells in *MHM1* locus in chicken. UCSC RefSeq gene models for galGal6 are shown. CAGE track shows only uniquely aligned reads (q>3 threshold). CAGE peak for *LOC112530614* was classified as a promoter. Cumulative expression of the MHM1 locus is female specific in chicken fibroblast cells.

Fig. S8 Autocorrelation of CAGE expression on chromosome Z. Genes expressed on chrZ in fibroblast cells were ordered by coordinates. Linear models for log(Fold change) values of compensated (FDR > 0.05) and non-compensated (FDR < 0.05) genes against coordinates on chrZ were submitted to Durbin-Watson autocorrelation test.

Fig. S9 Top male-female marker genes in avian fibroblast cells. Genes were sorted by FDR values. Top eight male and top eight female genes are shown for each species separately. Z-score calculated per gene on log2(TPM) values.

Fig. S10 *RPS6* is a universal male-biased marker. A: Upset plot for differentially expressed genes (FDR < 0.1) between male and female fibroblast samples defined by CAGE analysis. With such criteria *RPS6* is a top one gene in all studied bird species. **B:** RPS6 protein identity across bird species studied in this work. Zebra finch *RPS6* is relatively distant from other species and this result correlates with its gene expression profile: 5.8 folds up-regulation in zebra finch male samples, and 2-3 folds in other species (Figure 2C).

Fig. S11 *RPS6* expression in chicken embryonic cells and qPCR validation. A: *RPS6* expression in male and female chicken embryonic cells. Two scRNAseq datasets (see references in text) were realigned and recalculated using galGal6 assembly as a reference. Next, data was processed with Pagoda2 and Conos packages for R. We defined female cells as those with *HINTW* expression > 0. RPS6 expression in male cells was higher on all tested stages. Two-sided T-test with Benjamini-Hochberg correction applied for statistical estimates. **B:** Primer's efficiency calculation for *RPS6* and control genes *YWHAE* and *ARPP19*. Efficiency = (10^(-1/ Slope)-1)*100. RPS6 primer pair #2 and *YWHAE* were selected for qPCR analysis.

Fig. S12 Medium composition effect contribution on sex chromosome gene expression in fibroblast cells. A: PCA plots for top 5 components. Sample groups are circled when medium composition effect can be segregated by PCA. **B:** Top contributing genes to the selected medium associated component. Genes are marked by red color if shared at least by two species.

Fig. S13 Cross-species comparison of fibroblast gene expression. A-B: PCA for avian fibroblast cells. Using melGal5 and droNov1 assemblies (with low mapping ratio 42-54%) for turkey and emu, respectively (A). Using MGAL_WU_HG_1.0 and ZJU1.0 assemblies instead (mapping ratio 87-91%) (B). CAGE mapping ratio is critical for optimal cross-species analysis results. Gene model quality issue was overcome by additional CAGE peak classification and didn't affect the analysis. **C-F**: Cross-species comparison to mammalian

fibroblast cells. Birds were clustered into two groups – Galliformes and other (Duck, Zebra finch, Emu) (C-D). This result correlated with phylogenetic distances of the species (E). Differentially expressed genes between mammals and two bird groups are shown on panel (F).

Supplementary Figure 1

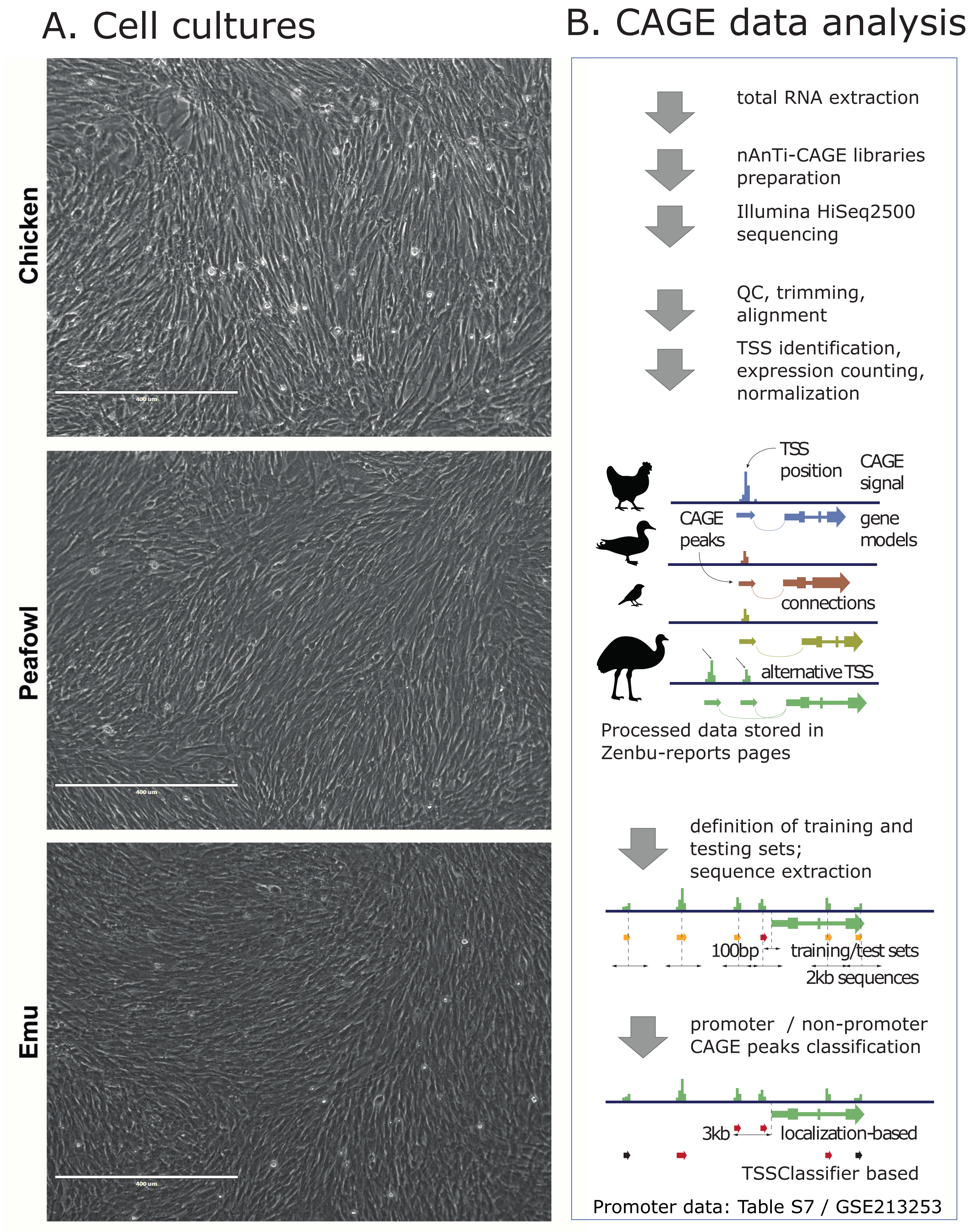

Supplementary Figure 2

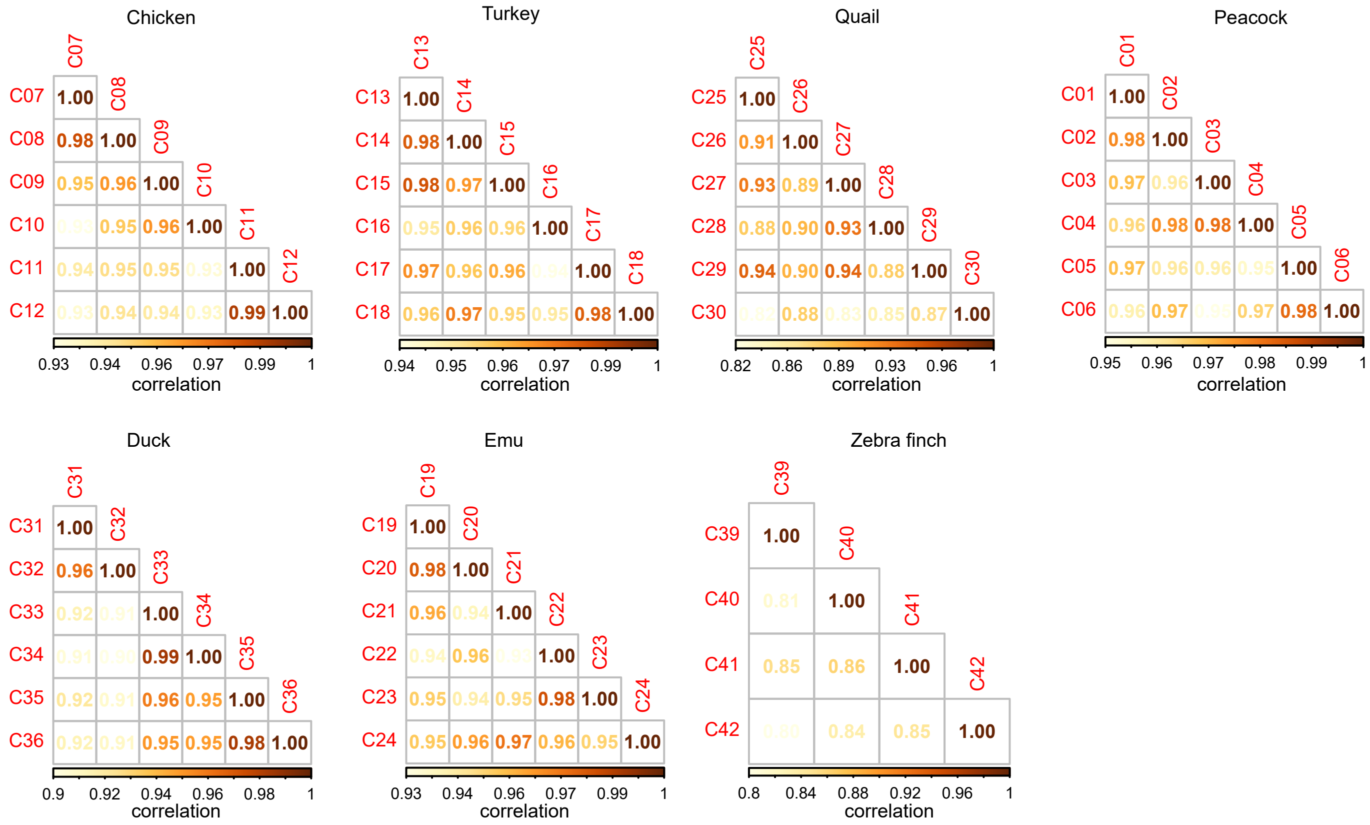

Supplementary Figure 3

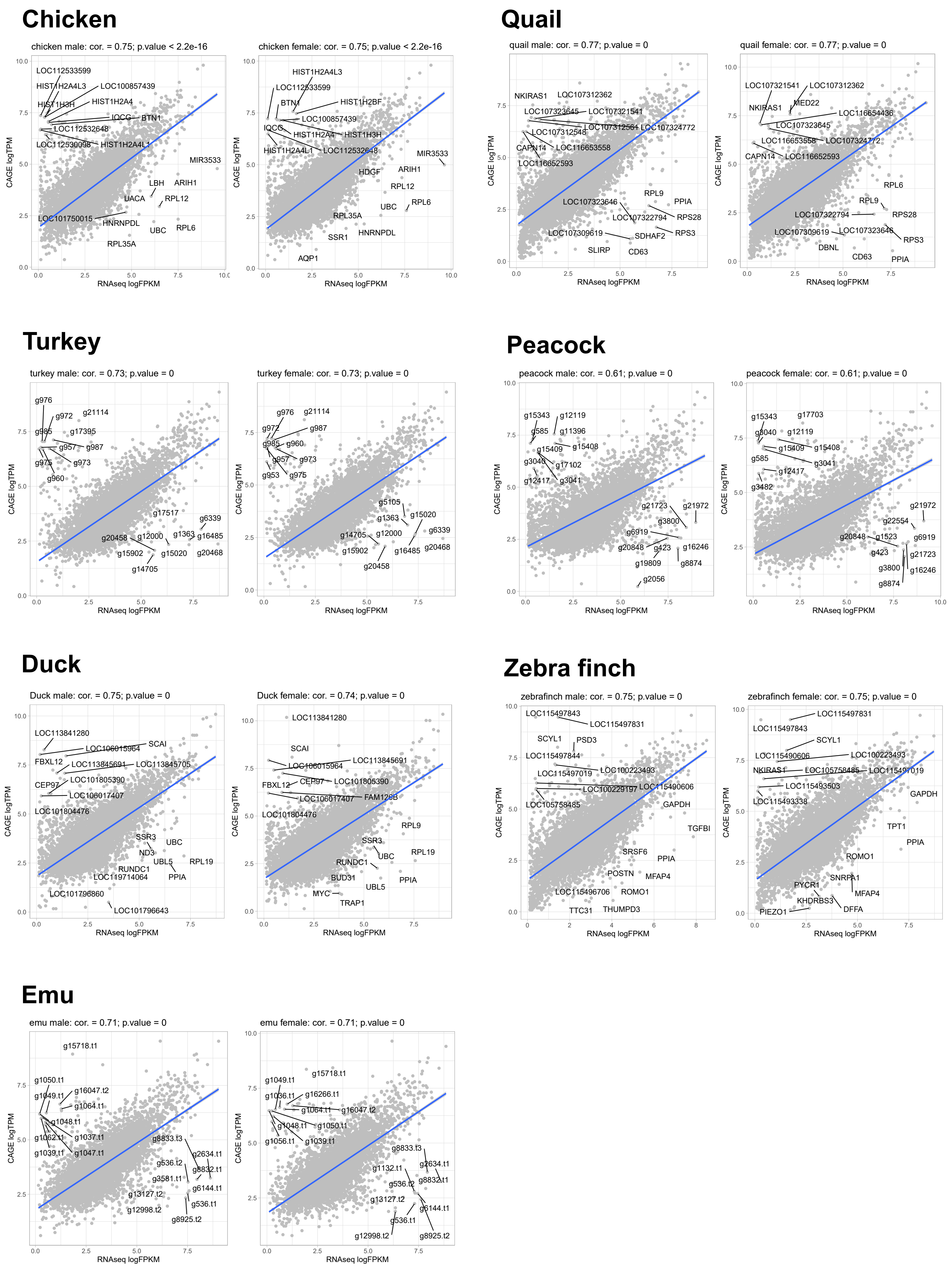

Supplementary Figure 4

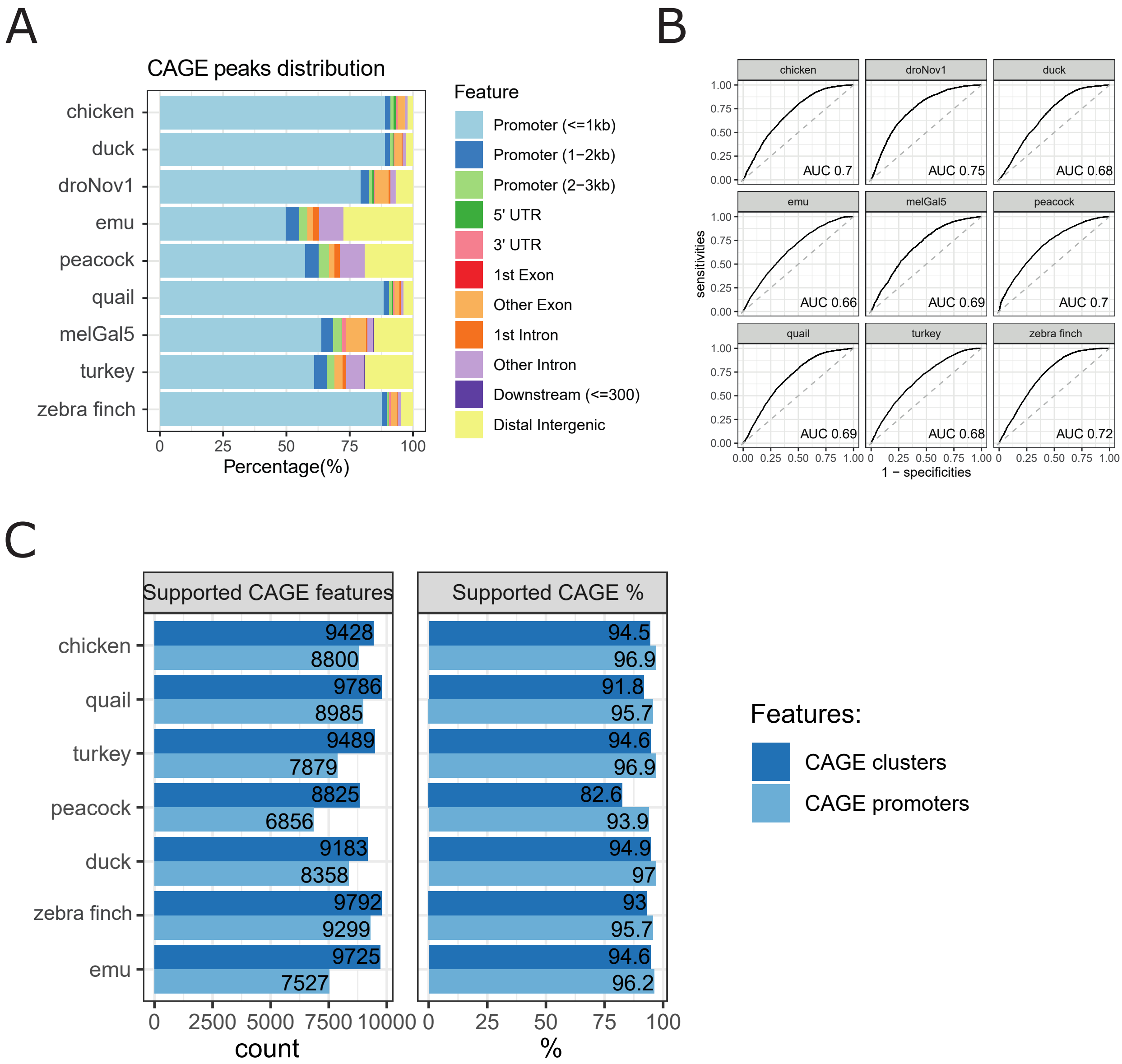

Supplementary Figure 5

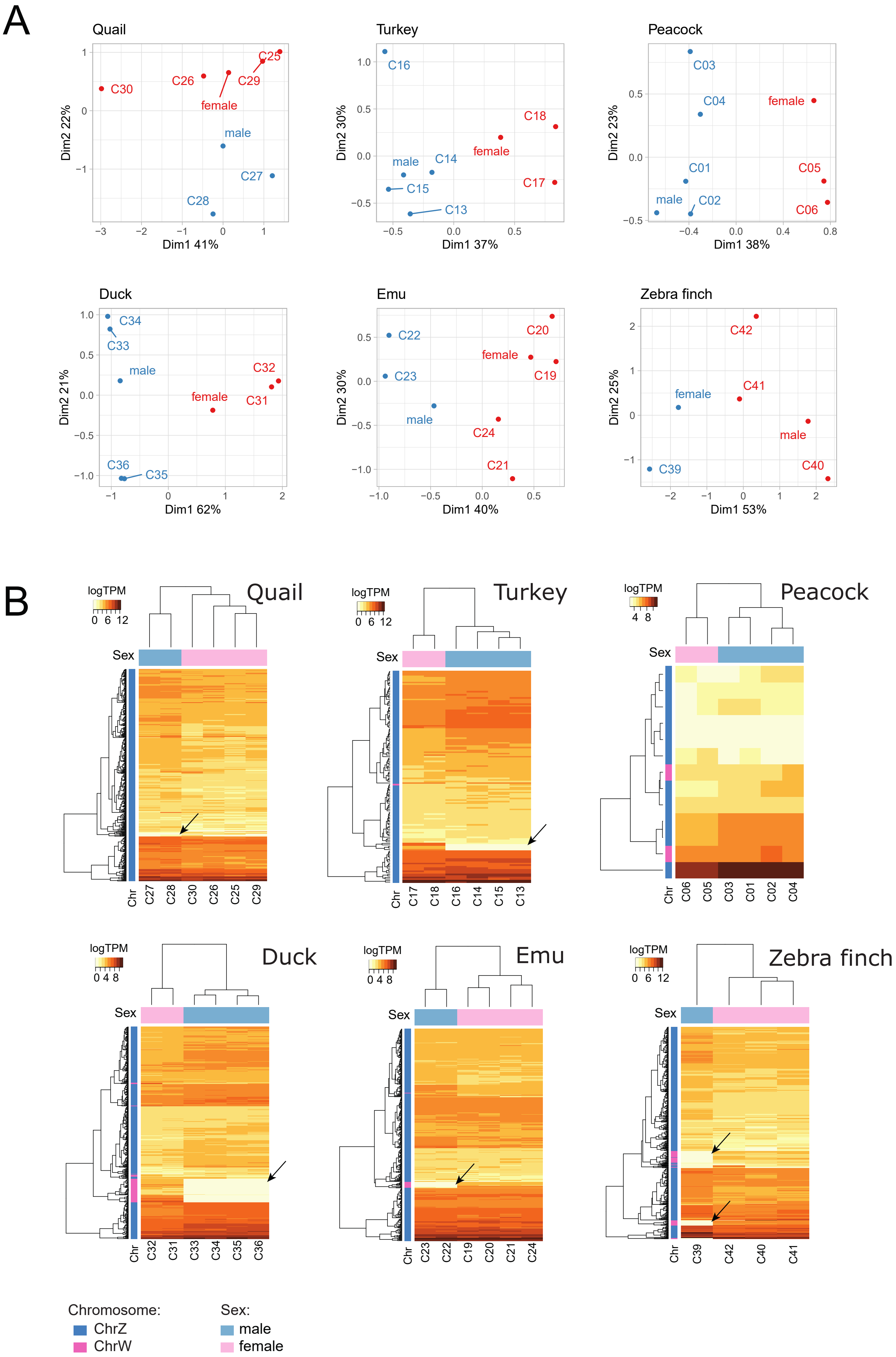

Supplementary Figure 6

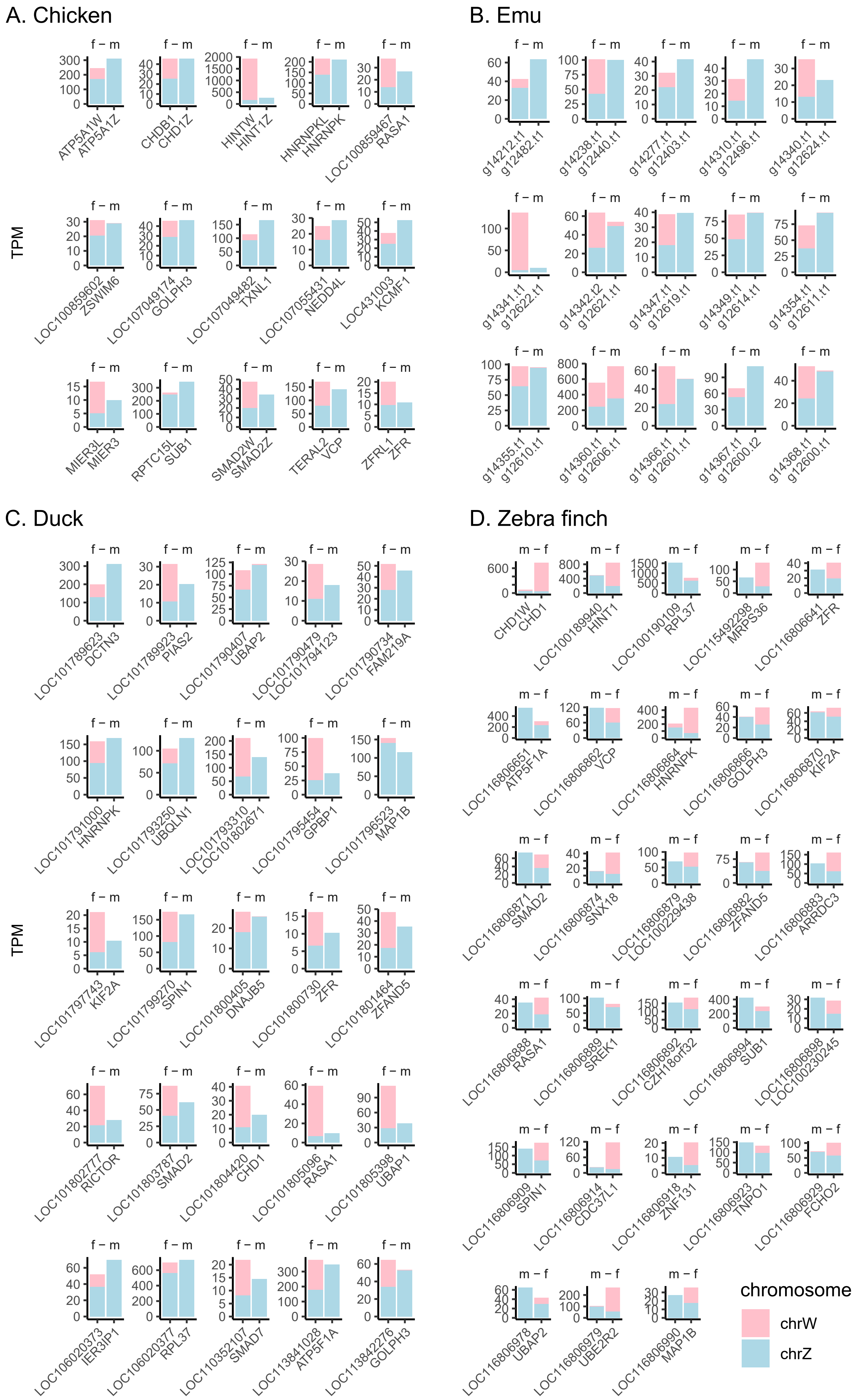

Supplementary Figure 7

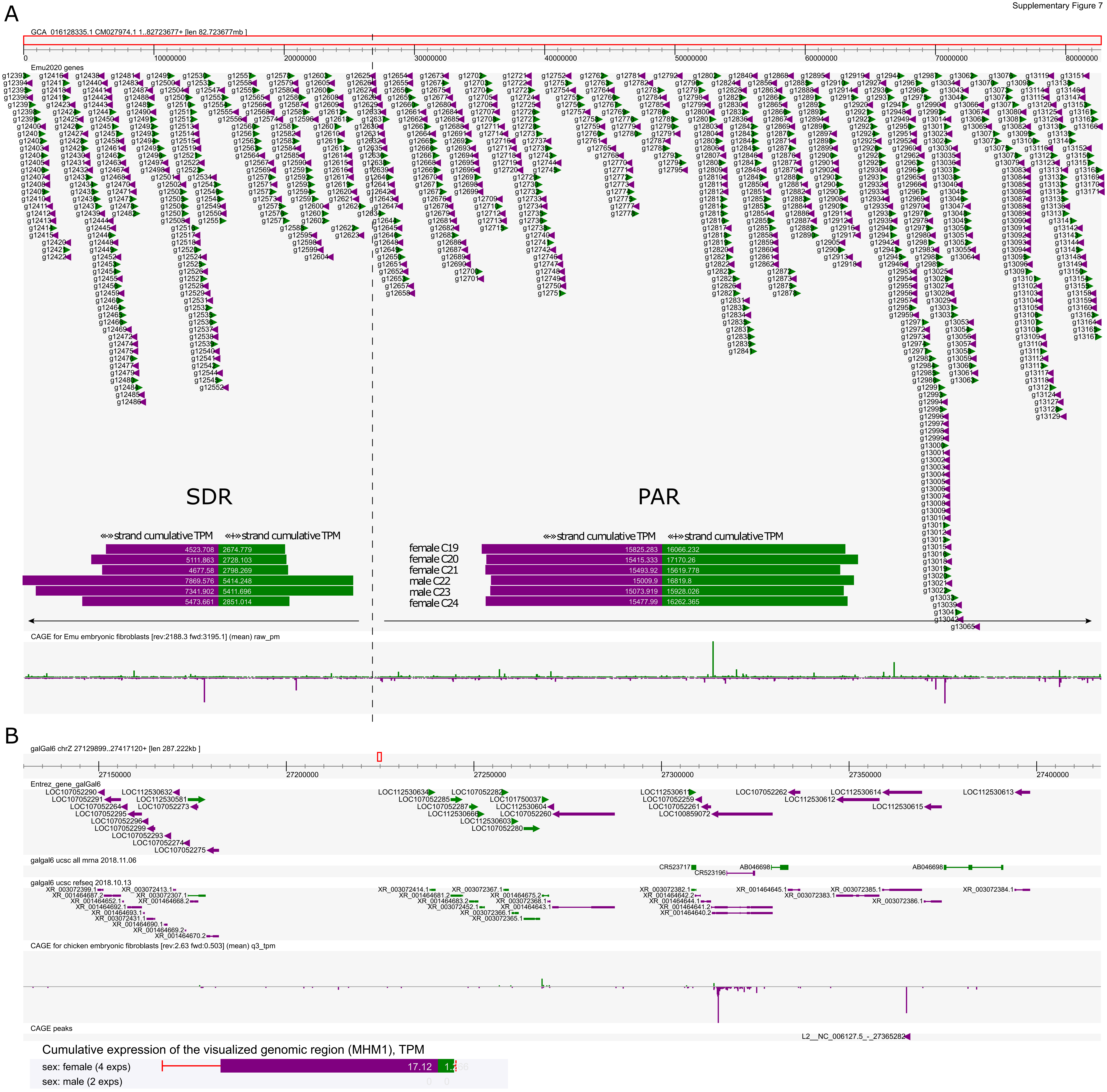

Supplementary Figure 8

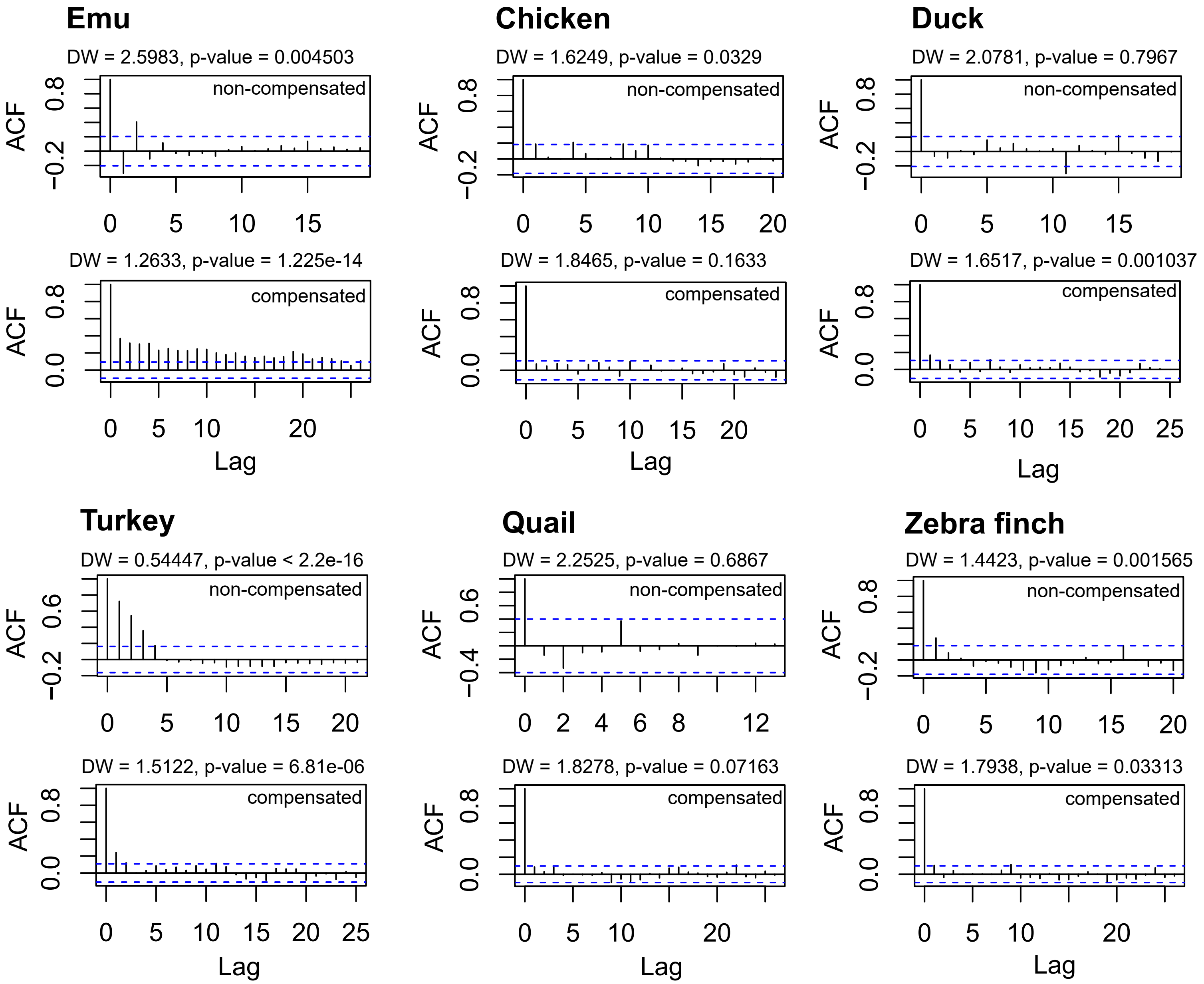

Supplementary Figure 9

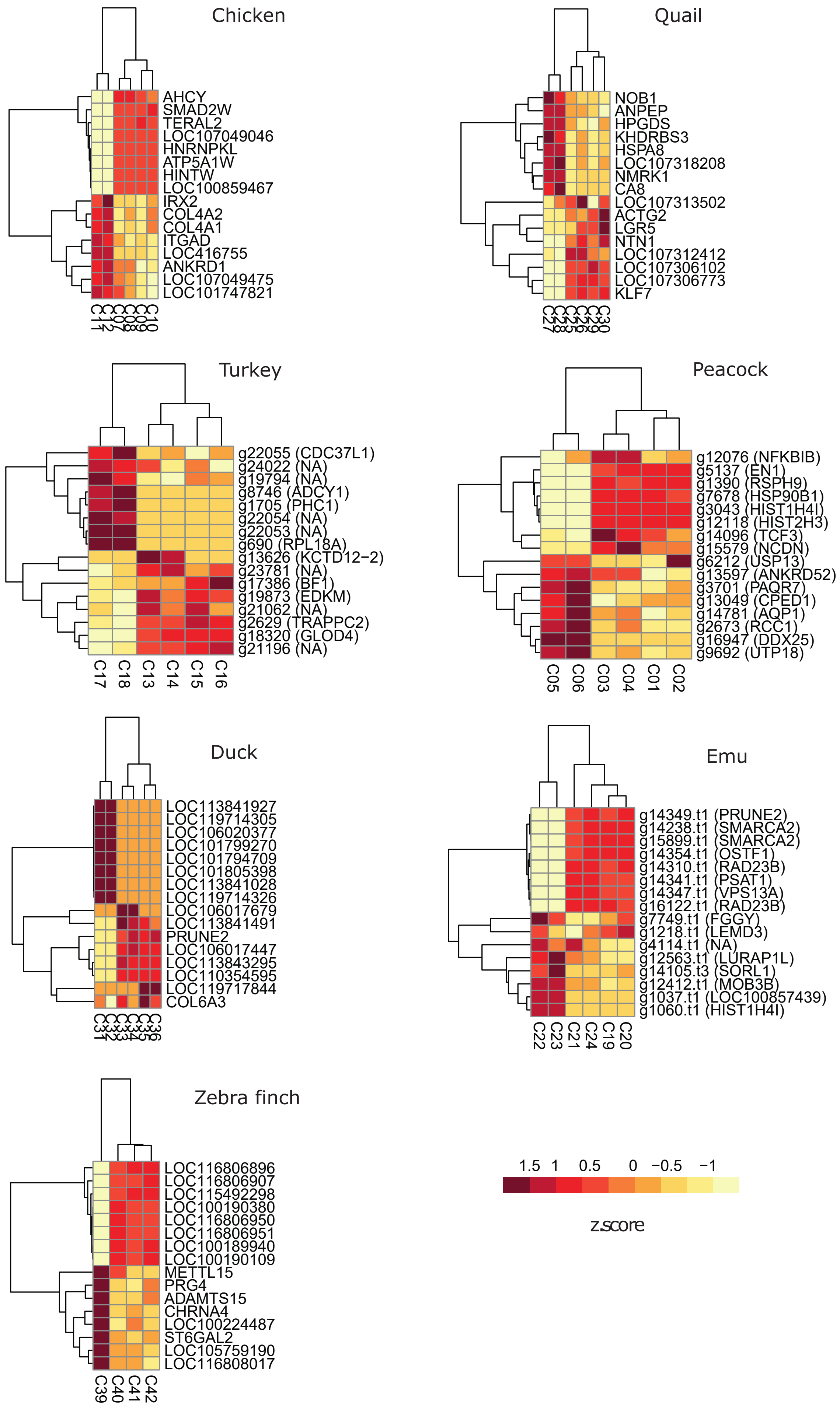

Supplementary Figure 10

A

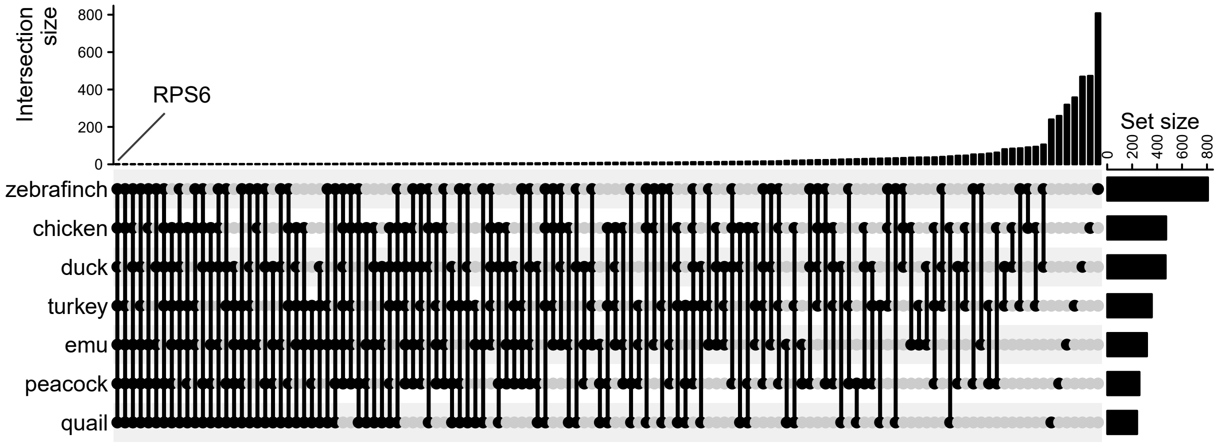

B

Precent identity

		1	2	3	4	5	6	7	8	9
Peacock	1		100,00	100,00	99,20	99,20	99,60	99,60	98,80	97,59
Duck	2	0		100,00	99,20	99,20	99,60	99,60	98,80	97,59
Emu	3	0	0		99,20	99,20	99,60	99,60	98,80	97,59
Mouse	4	2	2	2		100,00	98,80	98,80	97,99	96,79
Human	5	2	2	2	0		98,80	98,80	97,99	96,79
Quail	6	1	1	1	3	3		100,00	99,20	97,19
Turkey	7	1	1	1	3	3	0		99,20	97,19
Chicken	8	3	3	3	5	5	2	2		96,39
Zebra finch	9	6	6	6	8	8	7	7	9	

Differences

identity
high
low

Supplementary Figure 11

A

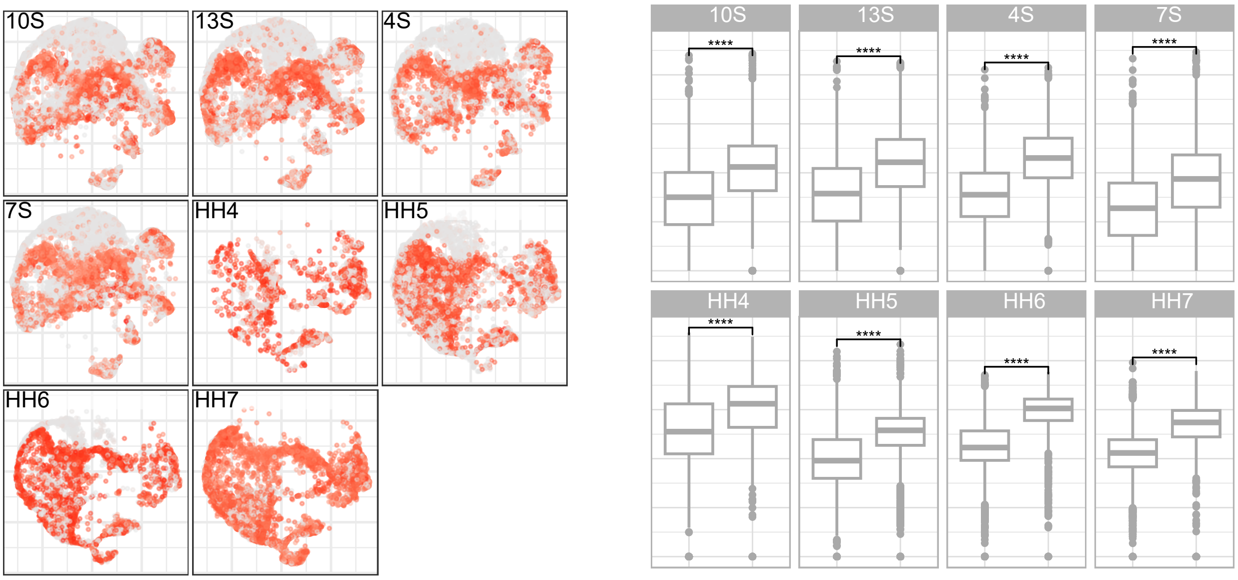

B

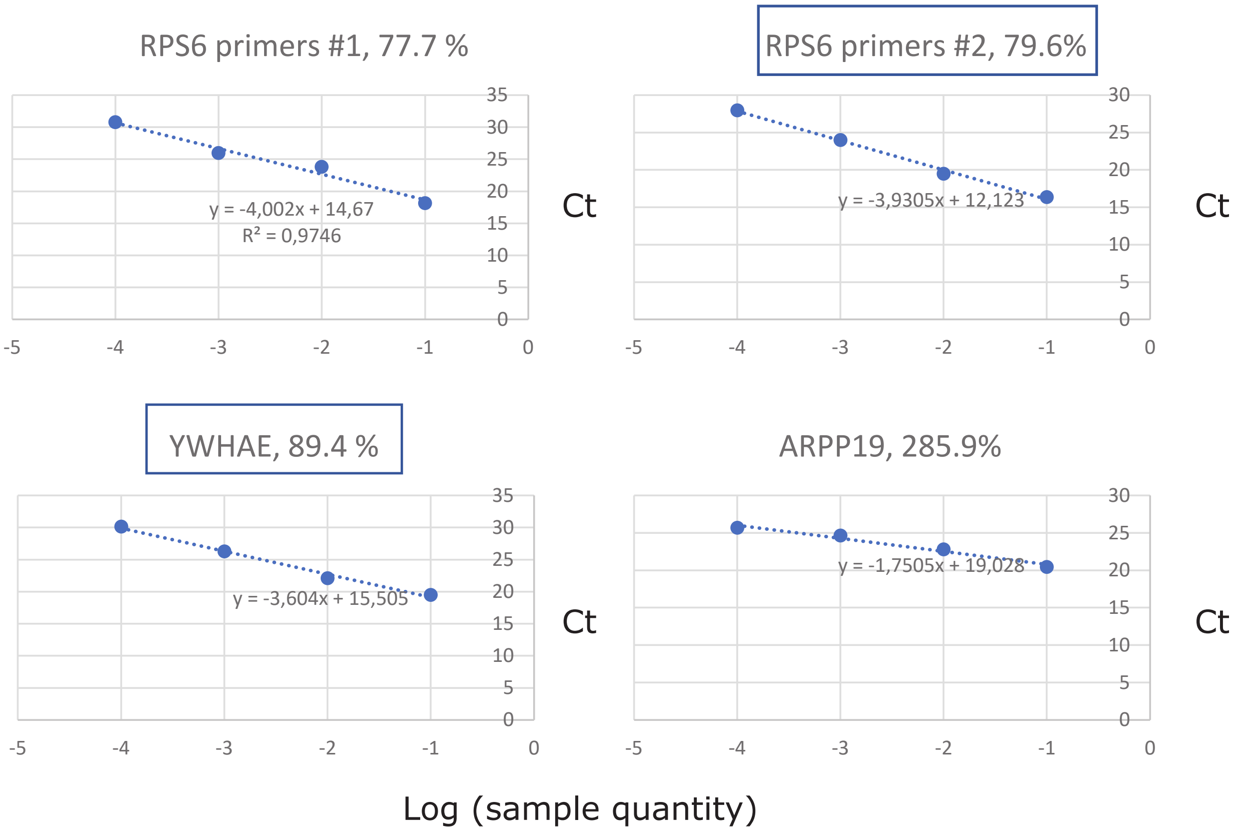

Supplementary Figure 12

A

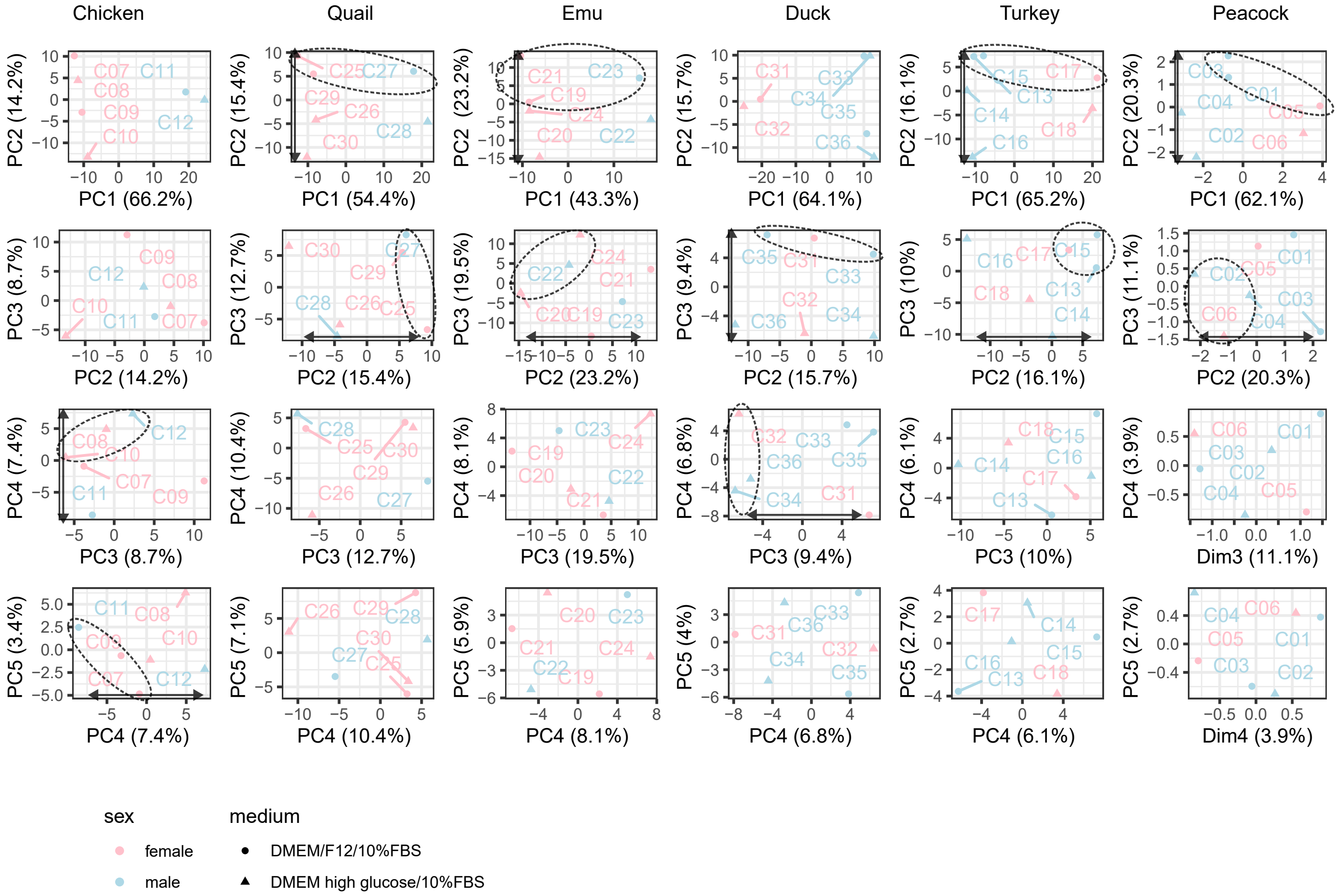

B

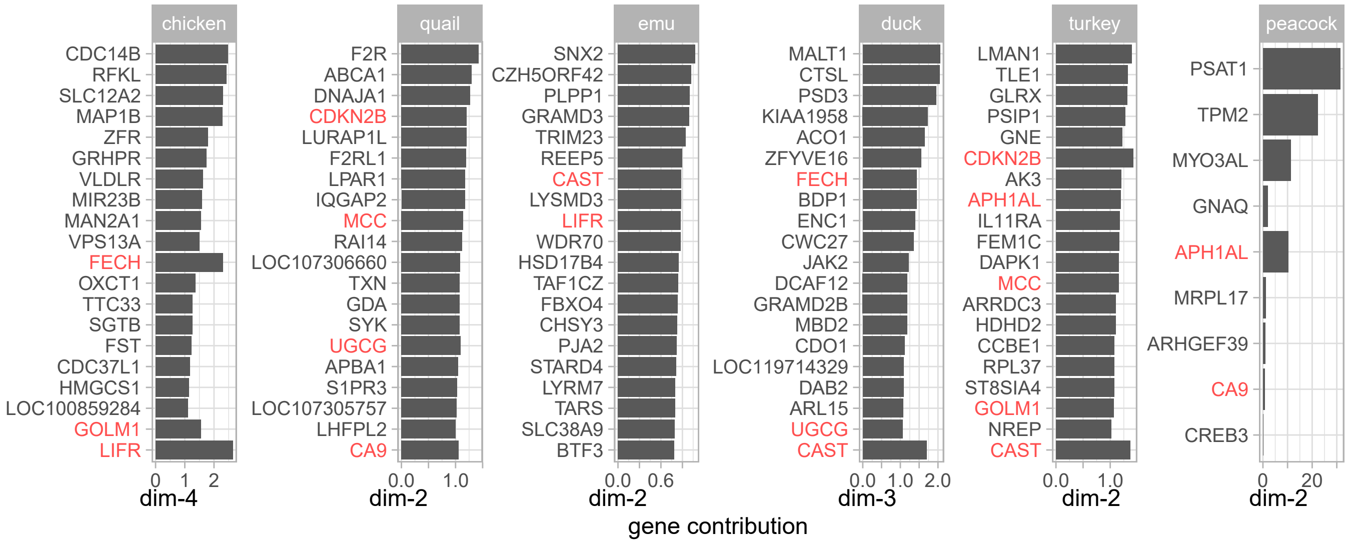

Supplementary Figure 13

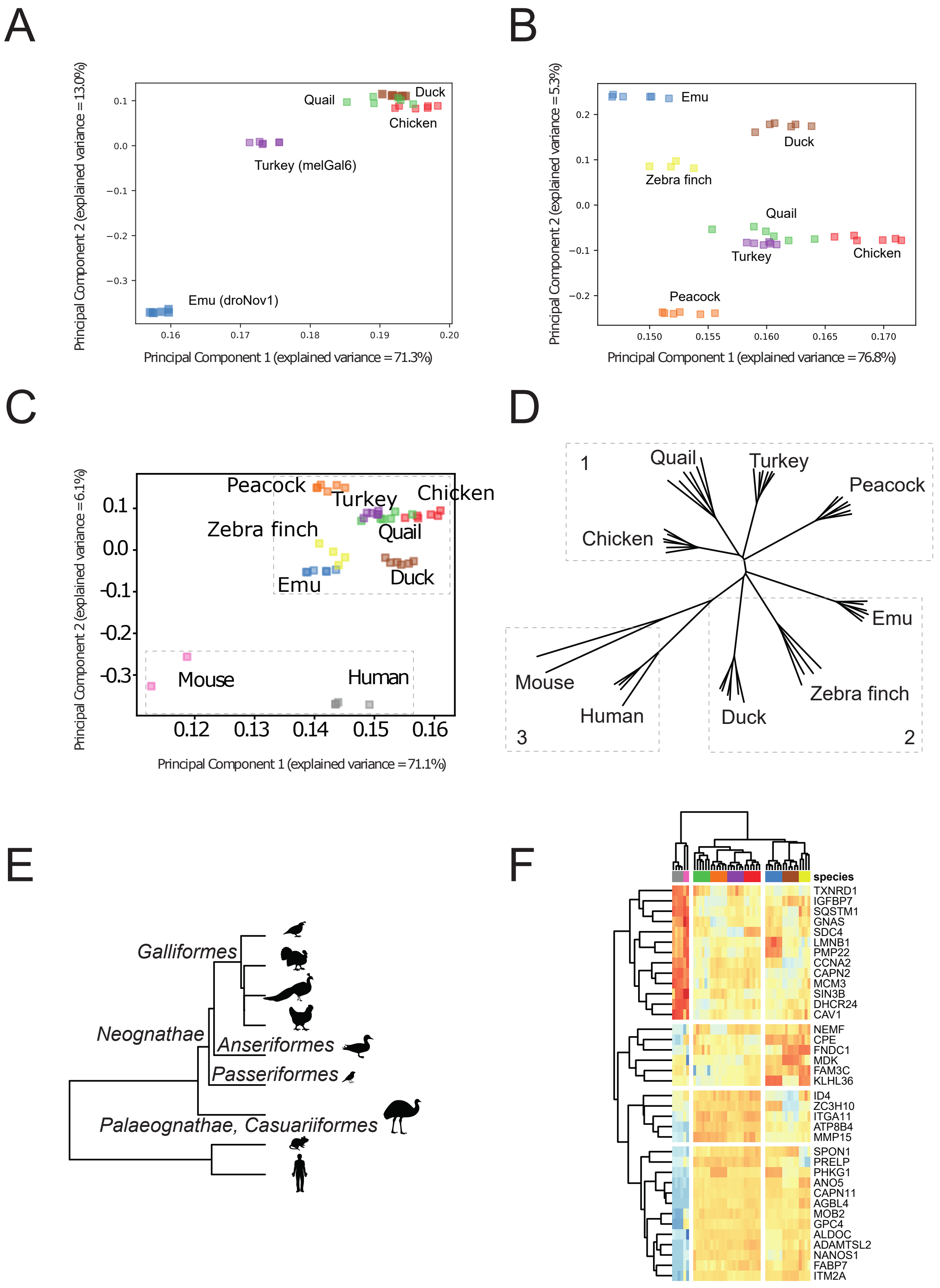

Supplement: Supplementary file 1 — Additional file 1: Fig. S1. Experimental design and the data analysis pipeline. Fig. S2. Correlation between CAGE replicates. Fig. S3. Correlation between CAGE-seq and RNA-seq methods in birds. Fig. S4. Peak distribution and model efficiency estimation. Fig. S5. Sample MDS plots and hierarchical clustering. Fig. S6. Expression profiles for gametologue genes defined by blast between chrW and chrZ genes. Fig. S7. Genomic views. Fig. S8. Autocorrelation of CAGE expression on chromosome. Fig. S9. Top male-female marker genes in avian fibroblast cells. Fig. S10. RPS6 is a universal male-biased marker. Fig. S11. RPS6 expression in chicken embryonic cells and qPCR validation. Fig. S12. Medium composition effect contribution on sex chromosome gene expression in fibroblast cells. Fig. S13. Cross-species comparison of fibroblast gene expression. [file 13059_2023_3055_MOESM1_ESM.pdf]
